# Microdissected Tissue vs. Tissue Slices—A Comparative Study of Tumor Explant Models Cultured On-Chip and Off-Chip

**DOI:** 10.3390/cancers13164208

**Published:** 2021-08-21

**Authors:** Dina Dorrigiv, Kayla Simeone, Laudine Communal, Jennifer Kendall-Dupont, Amélie St-Georges-Robillard, Benjamin Péant, Euridice Carmona, Anne-Marie Mes-Masson, Thomas Gervais

**Affiliations:** 1 Centre de Recherche du Centre hospitalier de l’Université de Montréal, (CRCHUM) and Institut du Cancer de Montréal, Montreal, QC H2X 0A9, Canada; dina.dorrigiv@polymtl.ca (D.D.); kayla.simeone@umontreal.ca (K.S.); laudine.eve.desreumaux@umontreal.ca (L.C.); jennifer.kendall-dupont.chum@ssss.gouv.qc.ca (J.K.-D.); amelie.st-georges-robillard@polymtl.ca (A.S.-G.-R.); benjamin.peant.chum@ssss.gouv.qc.ca (B.P.); euridice.carmona.chum@ssss.gouv.qc.ca (E.C.); 2 Institute of Biomedical Engineering Polytechnique Montréal, Montreal, QC H3T 1J4, Canada; 3 Department of Medicine, Université de Montréal, Montreal, QC H3T 1J4, Canada; 4 Department of Engineering Physics, Polytechnique Montréal, Montreal, QC H3T 1J4, Canada

**Keywords:** cancer treatment, drug screening assays, tumor explant culture platform, hypoxia, ex vivo model

## Abstract

**Simple Summary:**

Cancer drugs have the lowest success rate of approval in drug development programs. In order to address this, predictive preclinical assays that correctly reflect the clinical efficacy of a drug represent an urgent need in both clinical oncology and pharmaceutical research. To address this need, multiple tumor models have been developed, including tumor explant culture platforms, which are the only models that preserve the integrity of the tumor tissue. However, these models have not been fully characterized and multiple variables exist between studies. We investigated the effect of tissue size and culture vessel type on the survival of tumor explants by comparing micro-dissected tumor tissue with corresponding tumor slices. Our results show that the model geometry and culture vessel affect proliferation, apoptosis, and hypoxia in tissue cultures, and must be considered in designing tumor explant culture platforms.

**Abstract:**

Predicting patient responses to anticancer drugs is a major challenge both at the drug development stage and during cancer treatment. Tumor explant culture platforms (TECPs) preserve the native tissue architecture and are well-suited for drug response assays. However, tissue longevity in these models is relatively low. Several methodologies have been developed to address this issue, although no study has compared their efficacy in a controlled fashion. We investigated the effect of two variables in TECPs, specifically, the tissue size and culture vessel on tissue survival using micro-dissected tumor tissue (MDT) and tissue slices which were cultured in microfluidic chips and plastic well plates. Tumor models were produced from ovarian and prostate cancer cell line xenografts and were matched in terms of the specimen, total volume of tissue, and respective volume of medium in each culture system. We examined morphology, viability, and hypoxia in the various tumor models. Our observations suggest that the viability and proliferative capacity of MDTs were not affected during the time course of the experiments. In contrast, tissue slices had reduced proliferation and showed increased cell death and hypoxia under both culture conditions. Tissue slices cultured in microfluidic devices had a lower degree of hypoxia compared to those in 96-well plates. Globally, our results show that tissue slices have lower survival rates compared to MDTs due to inherent diffusion limitations, and that microfluidic devices may decrease hypoxia in tumor models.

## 1. Introduction

A critical bottleneck in pharmaceutical and clinical oncology is predicting the response of patients to anticancer drugs. Only 3.4% of new cancer drugs reach regulatory approval, despite the fact that oncology accounts for more than 40% of all drug development programs [1]. Cancer progression is due to an accumulation of multiple genetic and epigenetic alterations, creating intra- and inter-tumoral heterogeneity [2,3]. This complexity results in the ability of cancer cells to acquire innate and adaptive responses to different drugs, rendering the drug development and clinical decision-making uncertain even when predictive markers exist [4]. To address this variability, researchers have developed multiple preclinical models to evaluate the chemosensitivity profile of patients. In vitro 2D models, such as monolayer cultures of tumor-derived cell lines are simple and high-throughput, but they do not include the tumor architecture or interactions between cancer cells and the tumor microenvironment. This can result in discrepancies in treatment sensitivity compared to the parental tumors. To circumvent these drawbacks, a growing number of 3D models have emerged to incorporate 3D cell interactions and mass transfer limitations, which are more time- and cost-effective. The most commonly used 3D models are tumor spheroids because they are easy to form and have very high viability [5,6,7,8,9]. However, spheroids also lack the native tumor tissue arrangement and tumor-associated microenvironment. Patient-derived xenografts (PDX) are the current gold standard for in vivo tumor models and incorporate many elements of the primary tumor. However, the engraftment procedure can have an extremely low success rate (<10% for some cancers) and the long time frame for tumor development exceeds a clinically relevant time frame requirement for patient management [10,11]. A more recent group of 3D models are organotypic tumors, such as patient-derived tumor organoids and ex vivo tumor explants, which offer the possibility to preserve the tumor heterogeneity and the genomic and transcriptomic factors of each tumor [12,13,14]. Patient-derived tumor organoids require tumor deconstruction, cell purification, expansion, and tumor reconstruction steps, which destroy the original tumor architecture, and are time-consuming and labour-intensive. In contrast, ex vivo tumor explants do not require such processing and preserve the tumor integrity, including the cell heterogeneity and the specific tumor microenvironment [15,16,17].

The use of tumor explants, also known as precision cut or organotypic tissue slices, has steadily increased because they have been shown to replicate characteristics and chemosensitivity profiles of in vivo patient tumors [18]. However, the main challenges faced by tumor explant culture platforms (TECPs) are tissue viability in the absence of an intact vasculature system and the low throughput of drug screening assays due to limited tumor material. In response, several techniques and tools such as fortified culture media [19], shaker incubators [20], porous tissue lifts [21], and perifusion-based culture systems [22,23,24,25] have been evaluated to improve the short-term tissue viability. Moreover, the integration of a microfluidic infrastructure in TECPs has shown great potential to preserve tumor viability and increase the throughput via the reduced requirement of samples and reagents as well as the parallelization of experiments [26,27,28,29]. Our group has introduced a microfluidics-based TECP, known as the micro-dissected tissue (MDT) [30,31], in which MDTs with sizes similar to large spheroids can be generated rapidly using conventional tissue slicing and punching methods. Their small volume and roughly spherical shape make them easy to manipulate and amenable to high-throughput methods when originating from a primary tumor. This MDT methodology preserves the viability (>70% proliferative and <10% cell death) of tumor fragments for up to 15 days [31].

Despite an increasing number of publications describing tumor explants and their advantages over other 3D tumor models, there does not exist, to the best of our knowledge, any study which compares tumor explant culture strategies with each other on objective, experimental grounds. In this study, we compared the tissue viability of three TECP systems: (1) conventional culture of tissue slices in plastic 96-well plates; (2) the culture of tissue slices in oxygen-permeable microfluidic devices; and (3) the culture of MDTs in microfluidic devices. We investigated the effect of tumor tissue size and culture vessel type on ex vivo tissue survival to identify the limitations of these model systems in a non-perfused setting. Tissue slices (3 mm in diameter) and MDTs (500 µm in diameter) of the same thickness were produced from the same tumor specimen, while keeping the tissue volume and medium volume ratio constant. Samples were cultured for 15 days and examined at various time points for oxygen and glucose consumption, morphology, viability, proliferation, epithelial content, and hypoxia. Our results demonstrate that MDTs preserve higher levels of viability and proliferation than tissue slices over the same culture period. By comparing tissue slices in microfluidic devices and 96-well plates, we show that the oxygen-permeability of the microfluidic devices reduces the extent of hypoxia but does not prevent hypoxic cores from forming within the tissue. Our results identify the strengths and weaknesses of studied models and provide insights to orient the choice of tumor explant models for future studies.

## 2. Materials and Methods

### 2.1. Design and Fabrication of the Microfluidic Device

**Figure 1:**
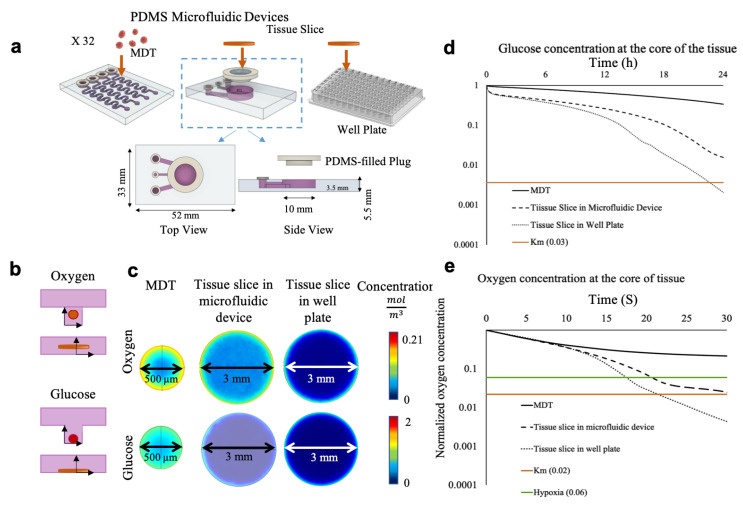
Design and operation of the microfluidic devices. (**a**) Schematic representation of the culture vessels. (**b**) Schematic representation of tissue positioning for worst case scenario for oxygen (top) and glucose (bottom). (**c**) Finite element simulation of the concentration distribution of glucose (at 24 h post-culture) and oxygen (at 30 s) in the midplanes of tumor models. Simulated changes in the concentration of glucose (**d**) and oxygen (**e**) in culture.

Microfluidic devices were composed of two polydimethylsiloxane (PDMS) layers obtained by moulding on micromachined polymethylmethacrylate (PMMA) or 3D-printed resin moulds. To form both layers, the elastomer base and the curing agent (Sylgard^®^ 184 silicone elastomer kit, Dow Corning, Midland, MI, USA) were mixed at a weight ratio of 10:1, degassed, and cured for 1 h at 80 °C. The MDT chip design has previously been described [31] and is illustrated in Figure 1a. Briefly, the bottom layer contained four fluidic channels of 0.9 × 1.1 mm rectangular cross-sections, each featuring an inlet and an outlet of 3.2 mm and 1.5 mm, respectively. The bottom layer consisted of a 4 × 8 array of 0.7 mm square cross-section wells that serve as traps for the MDT. The top layer of the tissue slice chip contained two fluidic channels that were 23 mm long with a 0.9 × 1.1 mm rectangular cross-section, each featuring an inlet of 3.2 mm in diameter. The bottom layer consisted of an 18 mm long fluidic channel with a 1.1 mm square cross-section connected to a 7 mm diameter cylinder, which served as the tissue slice chamber on one end and an outlet of 1.5 mm diameter on the other end. The top and bottom layers of both types of microfluidic devices were rendered hydrophilic and bonded using atmospheric plasma treatment to form enclosed channels. To seal the tissue slice chamber, a PDMS plug was designed from a 3D-printed mould (Figure 1a). All microfluidic devices and well plates were sterilized with ethanol and prepped with a triblock copolymer (Pluronic^®^ F-108, Sigma-Aldrich, St. Louis, MO, USA), as previously described [30].

### 2.2. Finite Element Methodology

We used COMSOL Multiphysics© software v.5.5 (COMSOL Inc, Burlington, ME, USA) to simulate the passive diffusion of oxygen and glucose in the MDTs and tissue slices in between the medium changes. The geometry of the model was drawn using built-in COMSOL drawing tools. The dimensions of the devices can be found in the Supplementary Material, Table S1. All simulations were conducted at a constant biological temperature (37 °C). Fick’s second law of diffusion was applied using the transport of diluted species physics in COMSOL to model the transport of oxygen and glucose in the culture medium and tissue. We first simulated oxygen transfer through the PDMS layers. As previously reported by Kim et al. [32], our simulations showed rapid oxygen exchange through the thin layers of PDMS at the top and bottom of the device that were exposed to ambient air. This can also be explained analytically. For instance, the effective diffusion time (t = x^2^/2D) for oxygen transfer through the 2 mm thick PDMS slab at the bottom of the device was less than 10 s, meaning that oxygen concentration through PDMS stabilizes after a few seconds. For this, we considered a constant oxygen concentration of oxygen at the top and bottom of the devices. However, oxygen transfer through other PDMS walls is not sufficient due to larger PDMS thicknesses and being constrained to medium-filled channels or wells. Hence, we imposed a no-flux (Neumann) boundary conditions at all other PDMS walls as well as plastic walls. All interface boundaries (Air/PDMS, PDMS/Medium and Medium/Tissue) were set with a continuity condition. We used Michaelis–Menten (MM) kinetics to model the glucose and oxygen consumption rates of the cells. The average Michaelis–Menten uptake kinetics found in the literature [30,33,34] imply high consumption rates in the abundance of nutrients and lower consumption rates when nutrients are depleted. The Michaelis–Menten constants served as the concentration thresholds, below which the normal cell metabolism is altered [35,36]. Furthermore, hypoxia is present in tumors when oxygen partial pressure falls below 10 mmHg (i.e., 13 µM dissolved oxygen in the culture medium) [37,38]. The design of the previously optimized MDT chip that ensures the high viability of MDTs [31,39] was adapted for tissue slices. We simulated the worst-case experimental scenario for glucose and oxygen consumption in tissue slices and MDTs: tissue settling on the bottom surface of the well, far from the medium-filled channels for glucose; and tissue floating in the middle of the well, far from the oxygen permeable PDMS walls for oxygen Figure 1b. Oxygen in microfluidic devices is replenished continuously and reaches steady state after a few seconds (30 s or less) in culture. In contrast, in plastic 96-well plates, it is a finite source and is depleted at the tissue core within seconds. Glucose is a finite source in all devices and is supplied through medium refreshment every 24 h. Tissue uptake and diffusion parameters are provided in the Supplementary Material, Table S2.

### 2.3. Ovarian and Prostate Cancer Cell Lines for Xenograft Production

Human carcinoma cell lines derived from ovarian cancer tumors, TOV112D (RRID:CVCL_3612) and TOV21G (RRID:CVCL_3613), and one metastatic prostate cancer tumor, DU145 (RRID:CVCL_0105), were used to produce mouse xenografts. Ovarian cancer cells were grown as monolayers (2D culture) in OSE medium (316-030-CL, Wisent Inc., Saint-Bruno-de-Montarville, Canada) supplemented with 10% fetal bovine serum (FBS; Gibco™, Thermo Fisher Scientific, Waltham, MA, USA), 55 mg/L gentamicin (Gibco™, Thermo Fisher Scientific) and 0.6 mg/L amphotericin B (Gibco™, Thermo Fisher Scientific). Prostate cancer cells were grown in RPMI medium (Thermo Fisher Scientific) supplemented with 10% fetal bovine serum (FBS; Gibco™, Thermo Fisher Scientific), 55 mg/L gentamicin (Gibco™, Thermo Fisher Scientific) and 0.6 mg/L amphotericin B (Gibco™, Thermo Fisher Scientific). After reaching confluency, cell suspensions (1,000,000 cells) were mixed with Matrigel (BD Biosciences, Franklin Lakes, NJ, USA) at a 1:1 ratio and subcutaneously injected into the flank of immunodeficient NOD.Cg-Rag1tm1Mom Il2rgtm1Wjl/SzJ female or male mice (Charles River Development, Wilmington, MA, USA), depending on the cancer type. Xenograft tumors were harvested once they reached a volume between 1500 and 2000 mm^3^. All animal procedures were performed in accordance with the Guidelines for the Care and Use of Laboratory Animals of the CRCHUM and approved by the Animal Ethics Committee (the Comité Institutionnel de Protection des Animaux).

### 2.4. MDT and Tissue Slice Production from Cell Line Xenograft Tumors

We adapted our previously published method [31] for the production of MDTs to prepare tissue slices. Briefly, a tissue chopper (McIlwain, Ted Pella©, Redding, CA, USA) was used to cut the xenograft into 350 µm thick tissue slices. Tissue slices were kept in Hank’s Balanced Saline Solution (HBSS, 311-516-CL, Wisent Inc., Saint-Jean-Baptiste, QC, Canada) supplemented with 10% FBS, 55 mg/L gentamicin and 0.6 mg L^−1^ amphotericin B. Tissue slices were further punched into MDTs using a 500 µm diameter tissue punch, and into standard tissue slices using a 3 mm diameter tissue punch (Zivic Instruments, Pittsburgh, PA, USA) and kept with the antibiotic and antifungal.

### 2.5. Tissue Loading, Trapping, and Culture of Tissue

The loading, trapping, and culturing of MDTs was performed as previously described [31]. For tissue slices, the device plug was removed, and one tissue slice was placed in the HBSS-filled tissue chamber using tweezers under sterile conditions. The plug was then put back in place to seal the device. One tissue slice per well was transferred to 96-well plates. Culture media were changed right after tissue loading and every 24 h for tissue slices and MDTs.

### 2.6. Formalin Fixation and Paraffin Embedding Protocol and Tissue Staining

We followed our previously published on-chip paraffin embedding lithography (PEL) protocol to produce MDTMA blocks for MDTs and standard paraffin blocks for tissue slices [31]. Each paraffin block was sliced into 4 µm thick sections using a microtome. Each paraffin slice was placed on a TOMO^®^ hydrophilic adhesion slide (Matsunami, Bellingham, WA, USA). Paraffin sections underwent hematoxylin and eosin (H&E) staining as well as immunofluorescence (IF) staining to assess the presence of tumoral cells (cytokeratin 8/18 (CK8/18) and human-specific mitochondria), proliferation (Ki-67), cellular death (cleaved caspase-3, CC3), and the presence of hypoxia (carbonic anhydrase 9, CA-IX) in the tumor models. IF staining was performed using the BenchMark XT automated stainer (Ventana Medical System Inc., Tucson, AZ). Antigen retrieval was carried out with Cell Conditioning 1 (Ventana Medical System Inc; #950-123) for 90 min for all primary antibodies. Rabbit anti-CA-IX (1:1000) antibody (ab15086, Abcam, Cambridge, United Kingdom), mouse anti-CK8/18 (1:200) antibody (IR09461-2, Agilent, CA, USA), mouse anti-mitochondria (1:2500) antibody (ab92824, Abcam), mouse anti-Ki67 (1:500) antibody (9449, Cell Signaling Technology, Danvers, MA, USA), and rabbit anti-cleaved caspase-3 (1:200) antibody (9661, Cell Signaling Technology) were automatically dispensed. The slides were incubated at 37 °C for 60 min and secondary antibodies were incubated at room temperature on the bench. All sections were scanned with a 20 × 0.75 NA objective with a resolution of 0.3225 μm (bx61vs, Olympus, Toronto, ON, Canada).

### 2.7. Quantification of Immunofluorescent Staining

To quantify protein expressions using IF, we used VisiomorphDP software (VisioPharm, Hørsholm, Denmark) [40,41]. Briefly, the tissue core surface area was detected through the Dapi channel. The surface area of CK8/18 and human mitochondria positive cells was labeled and quantified in the tissue core to differentiate human epithelial tumor cells from murine and stromal cells. Proliferation was quantified by dividing the surface area of Ki67-positive tumor cell by the total surface area of the tumoral cells. CC3 staining was observed both in the nuclei and in the cytoplasm. CC3 and CA-IX cytoplasmic staining were quantified by dividing the surface area of positively stained cells by the surface area of the epithelial cells.

### 2.8. Statistical Analysis

Statistical analyses were performed in GraphPad Prism© version 8.0 using the non-parametric one-way ANOVA Kruskal–Wallis test and post hoc Dunn’s test, because the data were not normally distributed according to the D’Agostino and Pearson omnibus normality test, and the sample size for tissue slices was small. For each experiment, a minimum of 15 MDTs or 3 tissue slices were analyzed at each time point for each condition. All experiments were repeated at least three times (N = 3). All data are reported as the mean ± SEM unless otherwise stated. The reported *p*-values were generated using a post hoc test (Dunn’s test).

## 3. Results

### 3.1. Numerical Simulation Predicts Sufficient Oxygen and Glucose in MDTs and Deficiency in Tissue Slices

Our group has previously designed a microfluidic platform that sustained the viability of 32 MDTs over 15 days [31]. Similar to our MDT model system, a microfluidic device was designed that holds a single tissue slice with a volume equivalent to 32 MDTs (Figure 1a). Thus, a total tissue volume of 2.4 µL surrounded by 250 µL of medium volume was considered for all three models for both simulations and experiments. Glucose uptake simulations were then conducted to gain insight on glucose depletion in tissue models during the 24 h medium change intervals. Simulations suggested that the concentration of glucose available in tissue slices fell below the Michaelis–Menten threshold, indicating glucose deprivation, regardless of the culture platform (Figure 1c,d). In contrast, MDTs had sufficient glucose (Figure 1c,d) after 24 h of culture. Simulation results for oxygen depletion in tissue models revealed that complete tissue anoxia (C = 0.5 µM) was present in the core of tissue slices cultivated in 96-well plates (Figure 1c,e). In the oxygen-permeable microfluidic devices, slices fared better but still suffered hypoxia in the core (C = 1.2 µM) (Figure 1c,e). In MDTs, a mild oxygen depletion was calculated, resulting in a final concentration of 34.4 µM oxygen in the core, which was about eight times greater than the hypoxic threshold (C = 4.6 µM) at its core (Figure 1c,e). Overall, our simulations predicted that MDTs had sufficient glucose and oxygen availability, and that tissue slices would experience both oxygen and glucose deficiencies.

### 3.2. Tumor Models Preserve the Characteristics of the Primary Xenograft Tumor

**Figure 2:**
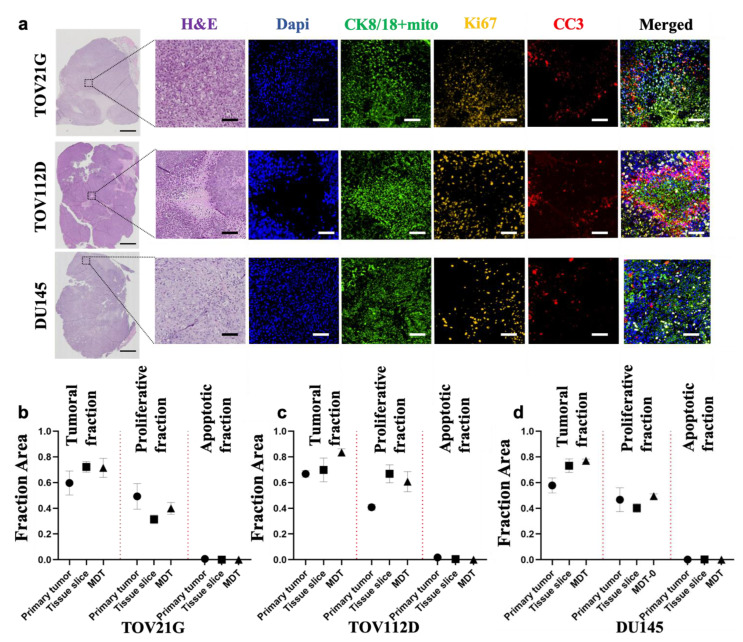
Characteristics of primary xenografts and tumor models of ovarian and prostate cancers. (**a**) Cross sections and staining of the entire primary xenograft tissues of ovarian cancer cell line (TOV21G and TOV112D) and prostate cancer cell line (DU145). Magnified tissue areas stained with H&E, tumor cell marker (CK8/18 + mito), proliferation marker (Ki67), and apoptosis marker (CC3). (**b**–**d**) IF scoring of primary xenograft and tumor models of TOV112D (**b**), TOV21G (**c**) and DU145 (**d**). All experiments were performed on the same xenograft as the starting material. Scale bars = 1 mm in whole tissue images and 100 μm in magnified images. Error bars = ±SEM.

To verify whether specific features of the primary xenograft tumor were captured in our tumor models, a fraction of the harvested specimen was instantly fixed in formalin and the remaining tumor tissue was used to produce the MDT and tissue slice tumor models. We performed histological and protein expression analyses to compare the primary xenograft tumor with the two tissue sizes (i.e., MDTs and tissue slices) on the day of harvest (day 0). H&E staining showed that the architecture of the primary xenograft tumor, including the varying cell-packing densities, tumoral structures and their spatial relation with stromal components, were preserved (Figure 2a). Furthermore, IF scoring of the tumor cells (i.e., CK8/18 combined with human mitochondria), proliferation (Ki67), and cell death (CC3) showed that the tumor models mirror the epithelial cellularity and viability of the corresponding primary xenograft tissue (Figure 2b–d). These results were consistent for the three different cell lines and suggest that, despite their differences in size, our tumor models represent the characteristics of the primary xenograft tumor, and that the tissue dissection procedure did not significantly alter tissue viability.

### 3.3. Viability and Proliferation Activity in MDTs Are Higher Than Tissue Slices over the Culture Period

**Figure 3:**
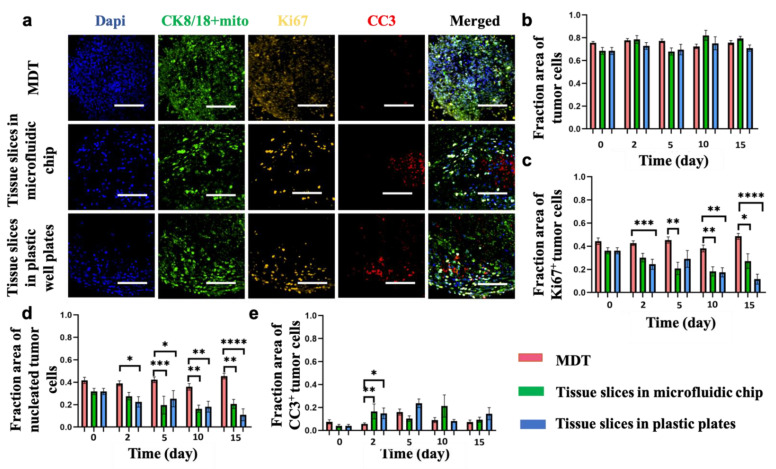
Tumor model viability over a 15-day culture period represented by ovarian cancer cell line xenografts (TOV21G). (**a**) Representative images of tumor models after 10 days in culture stained for nuclei (Dapi), human tumor cells (CK8/18+mito), cell proliferation (Ki67), and cell apoptosis (CC3). (**b**) IF scoring of tumor models showing stable expression of tumor-cell-specific marker (CK8/18+mito) over the 15-day period. (**c**–**e**) IF scoring of tumor models showing higher proliferation (**c**), larger nucleated area (**d**), and lower apoptotic cell death (**e**) in MDTs compared to tissue slices. Scale bars = 100 μm. Error bars = ± SEM. * *p* < 0.05; ** *p* < 0.01; *** *p* < 0.0001; **** *p* < 0.00001.

Changes in cell viability or tissue structure that occur over time in tumor explants may affect the interpretation of chemosensitivity analysis. Therefore, we examined the effect of culture conditions within the tumor models, specifically, variations in the cancer cell compartmentalization, and measured the fraction of intact nuclei (DAPI), apoptotic cells (CC3), and proliferative cells (Ki67) within the tumoral component after 0, 2, 5, 10, and 15 days in culture. H&E staining and tumor-cell-specific biomarkers (i.e., CK8/18 and mito) confirmed that the architecture of the primary tumor and the tumoral components were preserved in the tumor models for up to 15 days in culture (Figure 3a,b and Supplementary Figure S1a). Our observations suggest that MDTs and tissue slices have comparable proliferative activity in culture for a maximum of 5 days; however, tissue slices under both culture conditions had significantly lower proliferation compared to MDTs later in the culture period (Figure 3c). Moreover, we observed that a large number of nuclei were lost in tissue slices that were in culture for 10 days or more, which was also a sign of cellular death by various pathways [42,43]. We quantified the number of intact nuclei in the tumoral compartment of the tissue. IF scoring suggested that the area of cells with intact nuclei in the tumoral compartment in MDTs was significantly higher than tissue slices after 10 days in culture (Figure 3d). Furthermore, tumor cell proliferation and viability were generally lower in tissue slices cultured in 96-well plates compared to matched tissue slices in microfluidic devices. Analysis of the apoptotic marker CC3 showed no significant difference between the tumor models that had been in culture for 5 days or more (Figure 3e). The loss of CC3 signal could be due a high level of necrosis in tissue slices. These results are consistent for all the three cell lines used in this study (Figure S1). Taken together, our findings suggest that MDTs maintained higher viability and proliferation activity compared to the tissue slices over the culture period.

### 3.4. Elevated Levels of Hypoxia Are Found in Tissue Slices but Not in MDTs under Normoxic Culture Conditions

**Figure 4:**
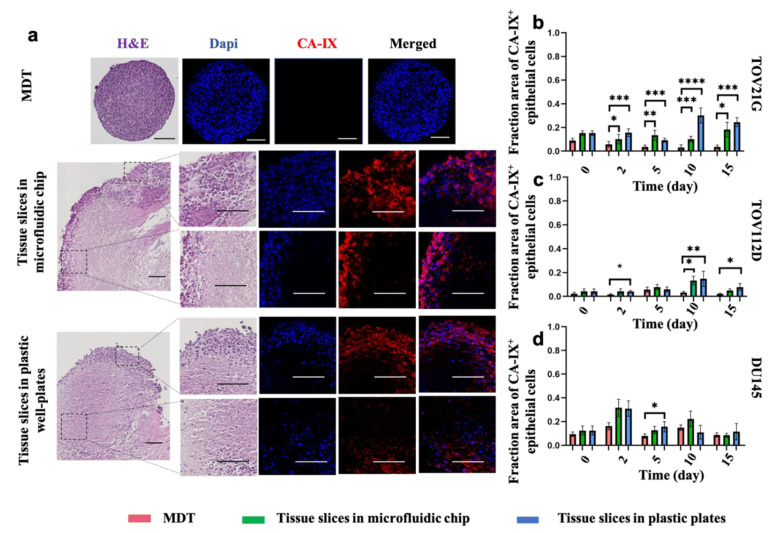
Incidence of hypoxia and necrosis in tumor models over a 15-day culture period using ovarian (TOV21G and TOC112D) and prostate (DU145) cancer cell line xenografts. (**a**) Representative images of tumor models produced from TOV21G after 10 days in culture stained with H&E or following IF with Dapi and CA-IX. H&E staining showed high levels of necrosis in tissue slices. b, c, and d IF scoring of tumor models of showed higher CA-IX expression over the 15-day period in tissue slices compared to MDTs. Results show increasing CA-IX expression until day 10 of culture in tissue slices for TOV21G (**b**), TOV112D (**c**), and DU145 (**d**) xenograft tumor models. Scale bars = 100 μm. Error bars = ± SEM. * *p* < 0.05; ** *p* < 0.01; *** *p* < 0.0001; **** *p* < 0.00001.

Ex vivo tissues develop hypoxic cores and subsequent necrosis in a non-perfused TECP, which may alter the drug screening analyses [9]. To date, no study has compared the extent of hypoxia and the hypoxia-associated cell death that occurs in different ex vivo tissue-derived models. To address this, we examined the incidence of hypoxia through carbonic anhydrase-IX (CA-IX) expression, a downstream target of the hypoxia-inducible factor 1-alpha (HIF1A) known to be up-regulated under hypoxic conditions [44,45,46]. We observed that tissue slices and MDTs had a comparable fraction of hypoxic cells at day 0. The expression level of CA-IX in MDTs was stable and generally lower than in tissue slices at different time points (Figure 4b–d). However, tissue slices under both culture conditions gained increasing and higher-than-MDT levels of hypoxia for up to 10 days in culture compared to their baseline hypoxic fractions (Figure 4b–d). In particular, higher levels of CA-IX expression were observed in tissue slices cultured in 96-well plates at all the time points. The reduction in CA-IX signal after 10 days in culture may be due to increased cell death at later time points (as shown in Figure 4e), resulting in the loss of signal. To further validate the loss of CA-IX signal after 10 days in culture, the H&E and IF staining of CA-IX (Figure 4a) were superimposed for pathological review and revealed that a large proportion of tissue slices in culture for 10 days or more had undergone coagulative necrosis, a form of necrosis mainly caused by hypoxia [43]. Furthermore, even though tissue slices produced from all the three cell lines showed higher levels of hypoxia and necrosis compared to their counterpart MDTs, the difference started earlier on and was more significant in ovarian cancer cell lines (i.e., TOV112D and TOV21G) compared to the prostate cancer cell line (DU145) (Figure 4b–d). This difference may be caused by the different metabolic profiles of cancer cell lines [47]. Furthermore, even though CA IX has been shown to be an excellent endogenous marker [48], it is reported to be less sensitive to prompt changes in the level of oxygenation (i.e., acute hypoxia or reoxygenation) compared to exogenous markers such as Pimonidazole [49,50]. Responses to exogeneous markers remain to be assessed. However, our transport model seems to match the CA IX results in all circumstances.

## 4. Discussion

The strong drive to improve the predictive power of model systems to maximize the chances of success in the clinic has made TECPs, be they tissue chunks, precision-cut tissue slices, or microdissected tissue, attractive in preclinical settings [19,29,30,51,52]. TECPs are the only model which preserve the native cellular diversity, tumor-stroma compartmentalization, and immune components found in primary animal and human tumors [53]. In addition to the biological aspects [54], there are time and cost gains in choosing TECPs over in vivo animal models. The use of primary patient tissue requires informed consent from patients, whereas patient tumor specimens are often readily available from surgery or biopsies with minimal or no additional risk for the patient [55]. Moreover, TECPs require fewer resources than in vivo models because many ex vivo tissue samples can be derived from a single specimen to test many drug conditions [56,57]. Of the two tumor formats and three culture conditions tested, microdissected tissue revealed themselves as the model of choice to maximize viability, throughput, multiplexing of treatment conditions, and overall process efficiency per unit tumor volume. Compared to tissue slices which showed clear signs of necrosis after 3–5 days, MDTs preserved high viability for a period of 15 days, which renders them useful for longer-term drug screening assays. We also found hypoxia, and consequently, hypoxia-induced cell death, to be significantly lower in microdissected tissue compared to tissue slices. Physically, this can be readily explained by the fact that there is a much greater surface-to-volume ratio in a sphere of a certain diameter than in a slice of the same diameter in thickness; thus, oxygen transport is enhanced in spheres [58].

Notably, however, tissue slices preserve larger tissue features in comparison to randomly selected MDTs from a tumor. Cutting tissue to small dimensions limits the use of tumor morphology and phenotypic evolution in treatment response prognoses. However, we have previously assessed the minimum number of MDTs that are required to represent a primary xenografted tumor by a Monte-Carlo simulation, which demonstrated that incorporating 15 or more MDTs in the study would address the question of heterogeneity [31]. Furthermore, randomly selected MDTs may even predict the heterogenous chemotherapeutic responses better than single tissue slices. Regarding hypoxia and oxygen gradients, one could argue that it plays a key role in tumor sensitivity and resistance to treatments [59,60] and must be present in a preclinical model. In this scenario tissue slice, being severely hypoxic and displaying sharp oxygen gradients may provide better prediction provided they can be kept from dying early in culture. However, the survival of tissues remains a problem which has not been resolved by device perfusion due to the very low tissue porosity and permeability [61,62], and perfusion techniques only increase the survival marginally [52,63]. Finally, microdissected tissue, although exhibiting little or no oxygen depletion, could always be used inside hypoxic chambers to test the treatment response as a function of oxygen availability. They form, as such, an interesting alternative to tissue slices [9,64]. However, the varying and fluctuating levels of oxygenation seen in vivo and the subsequent cell responses to such fluctuations cannot be modeled using these in vitro assays.

## 5. Conclusions

In this study, we investigated the effect of the tumor tissue size and culture vessel type on ex vivo survival by comparing micro-dissected tumor tissue with tissue slices cultured in PDMS-based microfluidic or plastic vessels. We observed that the viability and proliferative capacity of MDTs remained higher than those of tissue slices during the time course of the experiments. Moreover, oxygen-permeable microfluidic devices may improve the survival of tissue slices to some extent, but do not ultimately prevent the nutrient deficiency and cell death commonly associated with tissues of this size. We have shown that necrosis and hypoxia is preventable in MDTs but occurs in tissue slices under both culture conditions. However, tissue slices cultured in oxygen-permeable microfluidic devices have a lower degree of hypoxia compared to those in 96-well plates. Our results provide evidence that the model geometry and culture vessel play an important role in tissue survival and must be carefully selected in designing chemosensitivity assays.

## Data Availability

The data associated with the raw experimental data have been deposited in the Scholar Portal Dataverse: Microdissected tissue vs. tissue slices—A comparative study of two tumor explant models cultured on-chip and off-chip. (https://doi.org/10.5683/SP2/KNRWND, uploaded on 5 August 2021). All other data are contained in the article and supplementary material.

## Supplementary Materials

The following are available online at https://www.mdpi.com/article/10.3390/cancers13164208/s1, Figure S1: Tumor model viability over a 15-day culture period represented by ovarian (TOV112D) and prostate (DU145) cancer cell line xenograft models. Table S1: Dimensions of devices; Table S2: Tissue uptake parameters and diffusion properties.
