# Supplementary material for: Microdissected Tissue vs. Tissue Slices—A Comparative Study of Tumor Explant Models Cultured On-Chip and Off-Chip
Source: Cancers (Basel). 2021 Aug 21;13(16):4208. doi: 10.3390/cancers13164208 (PMC8394960; doi:10.3390/cancers13164208)
Supplement: Supplementary file 1 [file cancers-13-04208-s001.zip › 2021_Cancers_Dina_supplementary_19-06-2021.pdf]

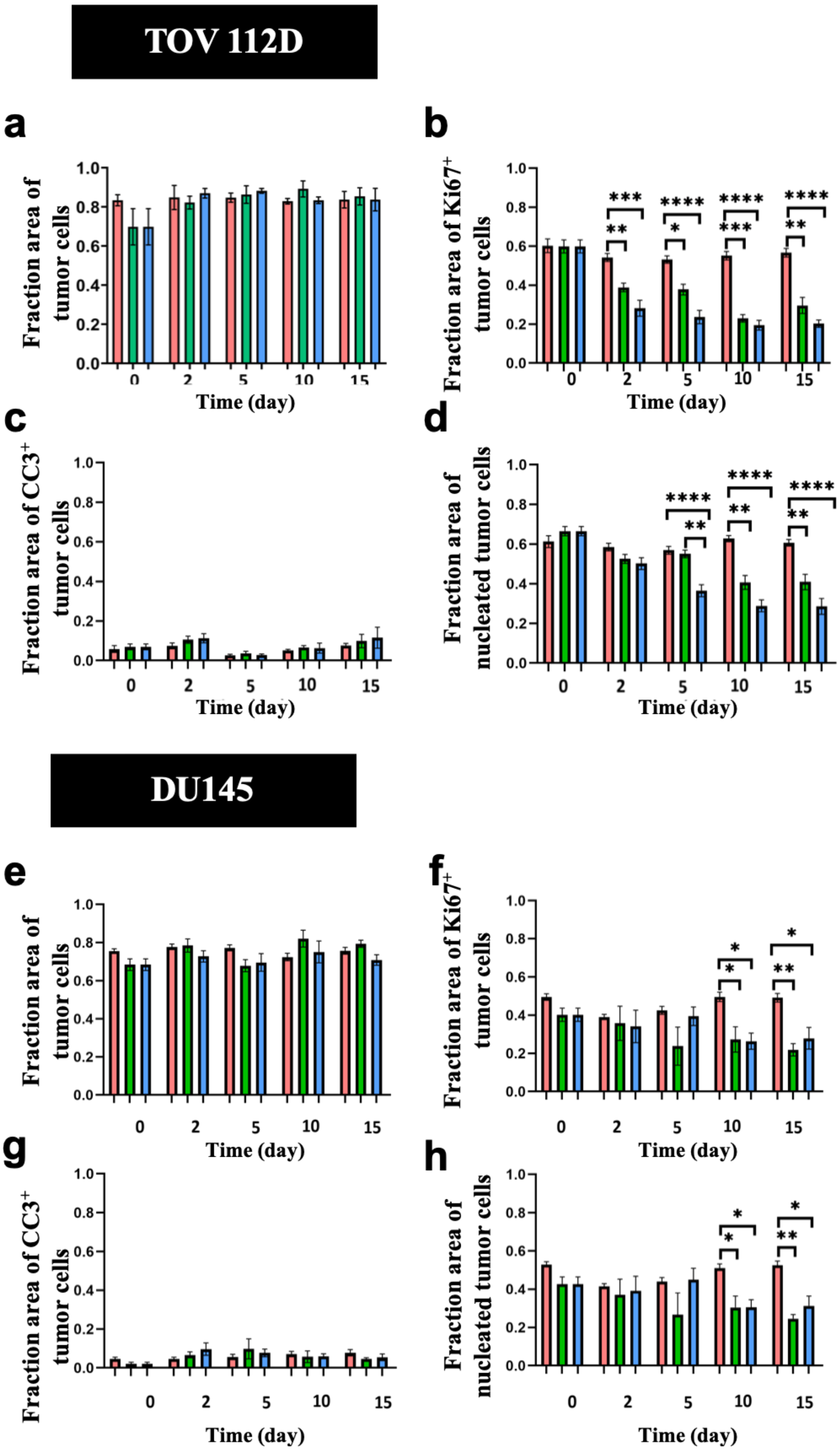

Figure S1) IF scoring of tumor model viability over a 15-day culture period represented by ovarian (TOV21G) and prostate (DU145) cancer cell line xenografts. tumor models showing comparable tumor cell compartment (a, e), higher proliferation (b, f), larger nucleated area (d, h), and varying apoptotic cell death (c, g) in MDTs compared to tissue slices. Scale bars = 100 µm. Error bars = ± SEM. *p<0.05; **p<0.01; ***p < 0.0001; ****p < 0.00001
