# Supplementary material for: Microdissected Tissue vs. Tissue Slices—A Comparative Study of Tumor Explant Models Cultured On-Chip and Off-Chip
Source: Cancers (Basel). 2021 Aug 21;13(16):4208. doi: 10.3390/cancers13164208 (PMC8394960; doi:10.3390/cancers13164208)
Supplement: Supplementary file 1 [file cancers-13-04208-s001.zip › Tables_Dorrigiv et al., Microdissected tissue vs tissue slices - A comparative study of two tumor explant models cultured on-chip and off-chip..pdf]

Supplementary tables

Microdissected tissue vs tissue slices - A comparative study of two tumor explant models cultured on-chip and off-chip.

Dina Dorrigiv[1,2], Kayla Simeone[1,3], Laudine Communal[1], Jennifer Kendall-Dupont[1], Amélie St-Georges-Robillard [1,4], Benjamin Péant[1], Euridice Carmona[1], Anne-Marie Mes-Masson[1,3] and Thomas Gervais[1,2,4] *

Table S1 dimensions of devices

**Parameter**	**value**		
Channel height (mm)	MDT chip	0.9	
	Tissue slice chip	Top layer	0.9
		Bottom layer	1.1
Channel width (mm)	MDT chip	1.1	
	Tissue slice chip	Top layer	1.1
		Bottom layer	1.1
Channel length	MDT chip	6	
	Tissue slice chip	Top layer	23
		Bottom layer	18
Tissue diameter (mm)	MDT	0.5	
	Tissue slice	3	
Tissue thickness (mm)	0.35		
Tissue chamber dimension (mm)	MDT well (cubical)	0.7	
	Tissue slice (cylindrical)	Diameter	7
		Height	2.5
	96 well-plate (Corning ®-cylindrical)	Diameter	6.4
		Height	17

Table S2 tissue uptake parameters and diffusion properties

<table>
<tr><th>Parameter</th><th colspan="3">value</th></tr>
<tr><td rowspan="2">Diffusion constant of glucose (cm^2/s)</td><td>Tissue</td><td colspan="2">$2.7x10^{-6}$</td></tr>
<tr><td>Medium</td><td colspan="2">$9.27x10^{-5}$</td></tr>
<tr><td rowspan="3">Diffusion constant of oxygen (cm^2/s)</td><td>Tissue</td><td colspan="2">$1.8x10^{-5}$</td></tr>
<tr><td>Medium</td><td colspan="2">$2.6x10^{-5}$</td></tr>
<tr><td>PDMS</td><td colspan="2">$3.4x10^{-5}$</td></tr>
<tr><td rowspan="4">Saturation concentration (mM)</td><td rowspan="3">Oxygen</td><td>Tissue</td><td>1.02</td></tr>
<tr><td>medium</td><td>0.21</td></tr>
<tr><td>PDMS</td><td>1.43</td></tr>
<tr><td>Glucose</td><td colspan="2">11</td></tr>
<tr><td rowspan="2">Partition coefficient</td><td>PDMS-Medium</td><td colspan="2">0.15</td></tr>
<tr><td>Medium-Tissue</td><td colspan="2">4.8</td></tr>
<tr><td rowspan="2">Maximum cellular uptake rate (mM/S)</td><td>Oxygen</td><td colspan="2">2.07</td></tr>
<tr><td>Glucose</td><td colspan="2">1.09</td></tr>
<tr><td rowspan="2">Michaelis-Menten constant (mM)</td><td>Oxygen</td><td colspan="2">$4.63x10^{-3}$</td></tr>
<tr><td>Glucose</td><td colspan="2">$4x10^{-2}$</td></tr>
</table>
